# Data of root anatomical responses to periodic waterlogging stress of tobacco (*Nicotiana tabacum*) varieties

**DOI:** 10.1016/j.dib.2018.09.046

**Published:** 2018-09-19

**Authors:** Hery Purnobasuki, Tutik Nurhidayati, Sucipto Hariyanto, Nurul Jadid

**Affiliations:** a Department of Biology, Faculty of Science and Technology, Airlangga University, Surabaya 60115, Indonesia; b Department of Biology, Institut Teknologi Sepuluh Nopember, Surabaya 60111, Indonesia

**Keywords:** Nicotiana tabacum, Periodic Waterlogging Stress, Anatomy of the roots

## Abstract

The data of root anatomical structure and the formation of aerenchyma tissues of five varieties of tobacco under waterlogging stress were obtained by modified paraffin method. Each tobacco varieties performed distinct anatomical adaptation response, including changes of cortical tissue, stele diameter, xylem diameter and the formation of aerenchyma under periodic waterlogging stress.

**Specifications table**

**Table:** 

Subject area	*Biology*
More specific subject area	*Anatomy plant biology*
Type of data	*Figures and text*
How data was acquired	*Periodic waterlogging Method, paraffin method, data and image analysis*
Data format	*Analyzed*
Experimental factors	*Five tobacco varieties were traited under periodic waterlogging stress for 14 days, including 7 days with waterlogging conditions and followed by 7 days treatment of flooding conditions.*
Experimental features	*Tobacco varieties used in this study include var. Jepon Palakean, Srumpung, Morakot, Somporis and Manilo. The observation of root anatomy was conducted using modified paraffin method.*
Data source location	*Department of Biology, Institut Teknologi Sepuluh Nopember, Surabaya, Indonesia*
Data accessibility	*The data are available with this article*

**Value of the data**

• The tobacco plant performs anatomical adaptation response of the roots under periodic waterlogging stress conditions through changes in cortical tissue, stele diameter, xylem diameter and the formation of aerenchyma.
• Data on root anatomical responses might be useful for further study on tobacco plant breeding.
• Data provided in this article could be combined with physiological and molecular study to elucidate the tobacco response mechanism against waterlogging and flooding stress.

## 1 Data

**Fig. 1:**
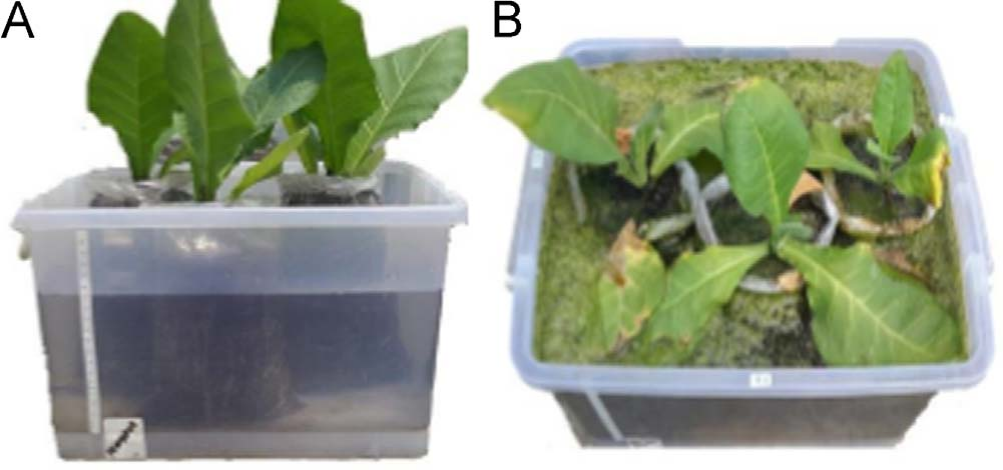
Treatment of periodic waterloging stress in Tobacco (*Nicotiana tabacum*): A.Waterlogging condition and B. Flooding condition.

**Fig. 2:**
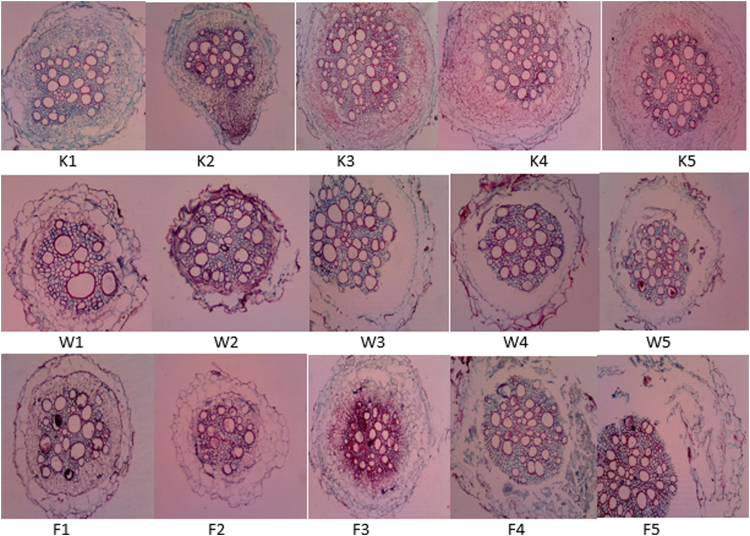
Cross-section of Fifth Root Varieties of Tobacco Plants under Periodic waterlogging (K: Tobacco Control Plants W: Tobacco Plants In *Waterlogging* Stress F: Tobacco Plants In *Flooding* Cash: 1. Var Jepon Palakean 2. Var Somporis3. Var, Marakot, 4. Var Srumpung, 5. Var Manilo).Observations were done using the Olympus CX 21 Light Microscope With Optilab Camera At Magnification (100×).

**Fig. 3:**
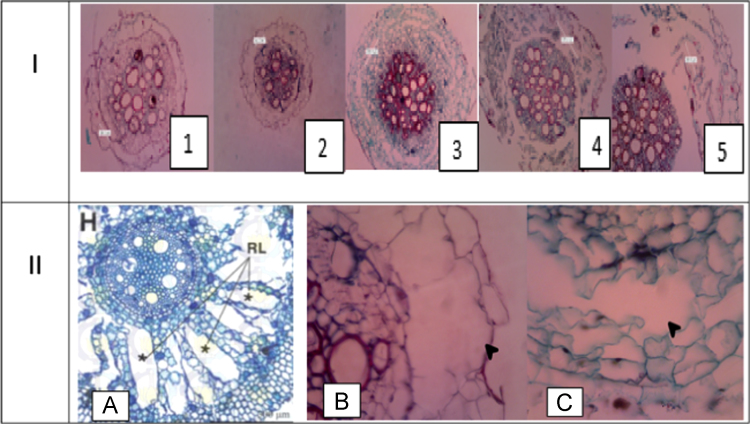
I: Aerenchyma In Cross Sliced Root of Tobacco Crops under periodic waterlogging stress Periodically / Flooding: (1. Var Jepon Palakean 2. Var Somporis; 3. Var. Marakot; 4. Var Srumpung; 5. Var.Marakot: Observations Using the Olympus CX 21 Light Microscope With Optilab Camera At 100× Magnification); II:II: A: *Typa latifolia* Root with radial type lisogeny [1] was used as literature standard;Arrows indicate aerenchyme: (B: aerenchyme Var Somporis And C: Var.Marakot: Observation Using the Olympus CX 21 Light Microscope With Optilab Camera At 400× Magnification).

The data on Fig. 1 shows the waterlogging stress treatment and Fig. 2 shows the cross-section of fifth variety of tobacco root under periodic waterlogging stress. Our data clearly showed the anatomical differences between treated plant and control. All of treated plants have bigger size of all root parameter and much number in aerenchyma, epidermal and endodermal cells. All varieties showed the formation of aerenchyma tissue after being treated with waterlogging and flooding stress (Fig. 2, Fig. 3). During treatment, tobacco varieties exhibited different root anatomical responses. Tobacco var. Jepon Palakean, Marakot and Manilo showed an increase of cortex thickness (more than 60% in waterlogging and more than 100% in flooding), diameter of stele and xylem (more than 75% in waterlogging and more than 40% in flooding). In contrast, var. Srumpung and Somporis exhibited a decrease of cortex thickness, diameter of stele and xylem (Table 1).

**Table 1:** Various root anatomical characters of Tobacco Varieties under periodic waterlogging stress.

Parameter	Treatment	Tobacco Plant Varieties				
		Palakean	Somporis	Marakot	Srumpung	Manilo
Root Diameter (µm)	W0	1356,9 ± 1,43ab	784,47 ± 0,05ab	1618,37 ± 0,36ab	1302,8 ± 0,23ab	1638,77 ± 0,27ab
	W1	1639,6 ± 0,43ab	705,93 ± 0,42ab	1585,53 ± 0,54ab	4410 ± 0,71ab	1274 ± 0,88ab
	F0	721,67 ± 0,30ab	1246,1 ± 0,48ab	1281,87 ± 0,45ab	1648,3 ± 0,17ab	1627,37 ± 0,42ab
	F1	1256,57 ± 0,22ab	846,4 ± 0,42ab	1470,33 ± 0,26ab	1421,4 ± 1,40ab	1670,13 ± 0,07ab
Stele Diameter (µm)	W0	484,13 ± 3,75ad	616,93 ± 0,36ad	981,7 ± 0,14ce	904,57 ± 0,11be	715,57 ± 0,91ce
	W1	833,33 ± 0,17be	562,83 ± 0,12ad	1016,57 ± 0,44ce	658,8 ± 0,21ad	892,67 ± 1,14ad
	F0	348,13 ± 0,19ad	721,67 ± 0,22ad	672,8 ± 0,13ad	842,07 ± 0,46ae	733 ± 0,37ad
	F1	760,07 ± 0,13bd	433,73 ± 0,49ad	751,33 ± 0,68ad	800,2 ± 0,60ad	1332,9 ± 0,52ce
Epidermal thickness(µm)	W0	71,43 ± 0,03bd	107,7 ± 0,05bd	171,9 ± 0,07bd	168,5 ± 0,02bd	177,4 ± 0,04bd
	W1	41,13 ± 0,33ac	28,23 ± 0,09ac	45,43 ± 0,19ac	35,53 ± 0,05ac	66,87 ± 0,23bd
	F0	71,43 ± 0,22bd	107,7 ± 0,10bd	171,9 ± 0,01bd	168,5 ± 0,12bd	177,4 ± 0,12bd
	F1	30,23 ± 0,01ac	23,73 ± 0,05ac	37,93 ± 0,06 ac	32,13 ± 0,09ac	31,23 ± 0,07ac
Cortex thickness (µm)	W0	158,2 ± 0,24ade	65,53 ± 0,17ad	79,13 ± 0,62ad	110,33 ± 0,17ad	155,8 ± 0,16ad
	W1	244,47 ± 0,21bde	42,57 ± 0,12ad	140,83 ± 0,37ad	108,9 ± 0,37ad	224,6 ± 0,04bde
	F0	86,27 ± 0,05ad	157,97 ± 0,22ade	201,7 ± 0,24bde	257,83 ± 0,58bde	225,37 ± 0,45bde
	F1	156,4 ± 0,39ade	155,27 ± 0,22ade	300,8 ± 0,61ce	249,53 ± 0,28bde	514,53 ± 1,14ce
Endodermal thickness (µm)	W0	156,03 ± 0,31bd	51,07 ± 0,20ac	227,03 ± 0,63bd	85,2 ± 0,22acd	120,5 ± 0,13ad
	W1	141,2 ± 0,21ad	50,17 ± 0,02ac	126,87 ± 0,63ad	172,73 ± 0,59bd	111,47 ± 0,15acd
	F0	44,67 ± 0,07ac	122,23 ± 0,10ad	92,27 ± 0,13acd	92,73 ± 0,34acd	207,7 ± 1,03bd
	F1	55,03 ± 0,34ac	45,4 ± 0,11ac	88,33 ± 0,04acd	53,9 ± 0,18ac	168,97 ± 0,44bd
Xylem thickness (µm)	W0	20,3 ± 0,02ab	30,8 ± 0,07ab	28,8 ± 0,03ab	28,73 ± 0,05ab	28,17 ± 0,03ab
	W1	29,7 ± 0,06ab	25,23 ± 0,05ab	36,5 ± 0,08ac	29,87 ± 0,05ab	30,47 ± 0,04abc
	F0	41,9 ± 0,04ac	35,33 ± 0,01ac	31,1 ± 0,03ab	30,37 ± 0,05ab	21 ± 0,06ab
	F1	35,37 ± 0,06ac	29,57 ± 0,01ab	36,9 ± 0,12ac	29,23 ± 0,01ab	34,67 ± 0,02ac
Xylem Diameter (µm)	W0	99,97 ± 0,03ad	116,8 ± 0,01bd	108,87 ± 0,02bc	135,27 ± 0,02bd	87,97 ± 0,18ac
	W1	121,17 ± 0,01bd	97,4 ± 0,02ac	148,1 ± 0,01bd	102,33 ± 0,07ad	116,2 ± 0,17ad
	F0	92,77 ± 0,02ac	102,37 ± 0,01ad	90,87 ± 0,01ac	102,27 ± 0,01ad	102,37 ± 0,01ad
	F1	119,53 ± 0,05bd	76,97 ± 0,03ac	93,6 ± 0,01ac	98,27 ± 0,02ac	87,03 ± 0,02ac
The number of aerenchyma cells	W0	3 ± 0,00ac	1 ± 0,01ab	2 ± 0,02ab	2 ± 0,01ab	3 ± 0,01ac
	W1	8 ± 0,05ac	5 ± 0,03ab	4 ± 0,02ab	5 ± 0,01ab	6 ± 0,02ac
	F0	2 ± 0,01ab	2 ± 0,02ab	1 ± 0,01ab	2 ± 0,01ab	1 ± 0,01ab
	F1	12 ± 0,01ad	10 ± 0,02ad	11 ± 0,02ad	8 ± 0,03ac	7 ± 0,00ac
Aerenchym Cell Length (µm)	W0	34,55 ± 0,06ac	41,4 ± 0,07ad	33,67 ± 0,05ac	45,62 ± 0,06ac	48,97 ± 0,03ac
	W1	115,16 ± 0,04be	97,23 ± 0,09bd	93,37 ± 0,03bd	123,17 ± 0,03be	80,93 ± 0,10ad
	F0	75,2 ± 0,02ad	45,32 ± 0,01ac	42,12 ± 0,03ac	82,45 ± 0,10ad	67,82 ± 0,02ad
	F1	137,76 ± 0,23be	138,63 ± 0,06be	166,1 ± 0,13bf	160,1 ± 0,45bf	168,17 ± 0,34be

Description: 1. numbers followed by the same letters in the same row and column for the measured parameters do not significantly different by Tukey Test at 5%; 2. Treatment Code: W0: Control 1; W1: Waterlogging; F0: Control 2; F1: Flooding

## 2 Experimental design, materials, and methods

### 2.1 Periodic waterlogging stress treatment

Tobacco seedlings aged 65 DAS (days after sowing) were grown under the periodic waterlogging condition in a plastic container measuring 40 cm × 30 cm × 20 cm (Fig. 1). Five tobacco varieties were used in this study including var. Jepon Palakean, Srumpung, Marakot, Somporis and Manilo. Periodic waterlogging stress treatment with a total 14 days was divided into 7 days in waterlogging conditions and 7 days under flooding conditions.

### 2.2 Sample preservation tobacco׳s roots

Root samples were firstly washed before being used for further analysis. Samples were prepared as ± 2 cm size. Subsequently, samples were fixed in FAA solution (formalin: acetic acid: 95% alcohol = 50 ml: 50 ml: 900 ml for every 1 l of solution) in a desiccator tool. Hydration process is then performed in a desiccator for 3 × 30 min. The FAA solution was then removed, and the sample was stored in a 70% alcohol solution [2].

### 2.3 Observation of root anatomical structure

The cross-sectional root anatomical structure was prepared using modified paraffin method. Procedures of the modified paraffin method used in this study were: (1) gradual dehydration with alcohol; (2) redehydration; (3) immersion through paraffin: dehydrant 1: 1;(4) Embedding;(5) Cutting using microtome;(6) Staining; (8) mounting using entelan. Root anatomical observation was conducted using light microscope with a camera Olympus CX 21 OPTILAB. Quantitative observations of root anatomical roots include total root diameter, stele diameter, epidermal thickness, cortical thickness, endodermic thickness, xylem thickness, xylem diameter, aerenchyme cell count and aerenchyme cell length. The data were analyzed by analysis of variance two way followed by Tukey test.
